# Plant expression of cocaine hydrolase-Fc fusion protein for treatment of cocaine abuse

**DOI:** 10.1186/s12896-016-0302-9

**Published:** 2016-10-19

**Authors:** Guojun Wang, Ting Zhang, Haifeng Huang, Shurong Hou, Xiabin Chen, Fang Zheng, Chang-Guo Zhan

**Affiliations:** 1 Molecular Modeling and Biopharmaceutical Center, College of Pharmacy, University of Kentucky, 789 South Limestone Street, Lexington, KY 40536 USA; 2 Department of Pharmaceutical Sciences, College of Pharmacy, University of Kentucky, 789 South Limestone Street, Lexington, KY 40536 USA; 3 Chemoinformatics and Drug Design Core, Center for Pharmaceutical Research and Innovation, College of Pharmacy, University of Kentucky, 789 South Limestone Street, Lexington, KY 40536 USA; 4 Present address: Harbor Branch Oceanographic Institute, Florida Atlantic University, 5600 US 1 North, Fort Pierce, FL 34946 USA

**Keywords:** Therapeutic protein, Fusion protein, Protein production, Drug abuse

## Abstract

**Background:**

A recently reported cocaine hydrolase (CocH3) fused with fragment crystallizable (Fc) region of human immunoglobulin G1, denoted as CocH3-Fc, is known as a promising therapeutic candidate for the treatment of cocaine overdose and addiction. A challenge for practical therapeutic use of this enzyme exists in the large-scale protein production and, therefore, it is interesting to identify a low-cost and feasible, sustainable source of CocH3-Fc production.

**Results:**

CocH3-Fc was transiently expressed in plant *Nicotiana benthamiana* leaves. The plant-expressed protein, denoted as pCocH3-Fc, was as active as that expressed in mammalian cells both in vitro and in vivo. However, compared to the mammalian-cell expressed CocH3-Fc protein, pCocH3-Fc had a shorter biological half-life, probably due to the lack of protein sialylation in plant. Nevertheless, the in vivo half-life was significantly extended upon the PEGylation of pCocH3-Fc. The Fc fusion did not prolong the biological half-life of the plant-expressed enzyme pCocH3-Fc, but increased the yield of the enzyme expression in the plant under the same experimental conditions.

**Conclusions:**

It is feasible to express pCocH3-Fc in plants. Further studies on the pCocH3-Fc production in plants should focus on the development of vectors with additional genes/promoters for the complete protein sialylation and for a better yield.

## Background

Cocaine is the most reinforcing drug in stimulating the reward pathway of the brain and teaching the user to take it again [6, 13, 15]. Despite decades of effort, classical pharmacological approaches to antagonizing neuropharmacological actions of cocaine has not proven successful, because it would be extremely difficult to antagonize its physiological effects without affecting normal functions of central nervous system (CNS) [29]. The inherent difficulties of antagonizing cocaine in the CNS led to the development of protein-based therapeutic agents that can tightly bind with cocaine and, thus, prevent cocaine from reaching the CNS or accelerate cocaine metabolism [21, 29]. In particular, the pharmacokinetic approach with an efficient cocaine-metabolizing enzyme is recognized as the most promising treatment strategy for cocaine overdose and addiction [2, 5, 20, 29]. Unlike the stoichiometric binding of an antibody with drug, one enzyme molecule can degrade multiple drug molecules, and the efficacy of an enzyme is dependent on its catalytic rate constant (*k*_cat_) and Michaelis-Menten constant (*K*_M_) against the drug.

The principal metabolic enzyme of cocaine in human body is butyrylcholinesterase (BChE) in the plasma, producing biologically inactive metabolites. However, the catalytic efficiency (*k*_cat_/*K*_M_) of wild-type human BChE against naturally occurring (−)-cocaine is too low (*k*_cat_ = 4.1 min^−1^ and *K*_M_ = 4.5 μM) [22] to be effective for (−)-cocaine metabolism. Below we will always refer cocaine to (−)-cocaine, unless explicitly stated otherwise, for convenience. In previous studies, we successfully designed and discovered human BChE mutants with at least 1000-fold improved catalytic efficiency against cocaine compared to wild-type human BChE, and these BChE mutants are recognized as *true* cocaine hydrolases (CocHs) in literature [2, 17, 25, 27, 28]. The first one of our designed CocHs, known as CocH1 (A199S/S287G/A328W/Y332G mutant of human BChE) [17, 26], was fused with human serum albumin (HSA) to improve the in vivo stability [2], and the obtained HSA-fused CocH1 is also known as Albu-CocH, Albu-CocH1, AlbuBChE, or TV-1380 in literature [2, 5, 20, 23]. Clinical trials demonstrated that TV-1380 is safe and effective for use in animals and humans ([5, 20]). TV-1380 has a biological half-life of ~8 h in rats [2] and 43–77 h in humans [5]. However, its actual therapeutic value for cocaine addiction treatment is still limited by the relatively lower catalytic activity of TV-1380 against cocaine compared to the more recently reported human BChE mutants, and the costs for large-scale protein production. The lower the catalytic activity of the enzyme against cocaine, the higher the required dose of the enzyme, and thus the higher the costs would be.

Notably, our more recently reported A199S/F227A/S287G/A328W/Y332G mutant of human BChE, known as CocH3 [24, 28], is significantly more active against cocaine compared to CocH1. Further, we have recently designed, prepared, and tested a long-acting form of CocH3, denoted as CocH3-Fc [4], a fusion protein in which the C-terminus of CocH3 is fused with the N-terminus of fragment crystallizable (Fc) region of human immunoglobulin G1 (IgG1). The CocH3-Fc protein expressed in Chinese hamster ovary (CHO) cells may be regarded as a catalytic antibody analog, because it is as active as the unfused CocH3 against cocaine with a considerably longer biological half-life (e.g. *t*_1/2_ = ~107 h in rats) like an antibody [4]. A single dose of CocH3-Fc was able to block cocaine-induced hyperactivity and toxicity for a long period [4].

On the other hand, it has been very challenging to express BChE and its mutants in a commercially feasible expression platform. For example, the low yield of protein expression in CHO cells equates with high costs for protein production and ultimately treatment. It is highly desired to identify a low-cost and feasible, sustainable source of CocH3-Fc production for practical application of CocH3-Fc-based enzyme therapy. Generally speaking, rapid, transient expression of a foreign protein in plant is readily scalable for large-scale production with low costs [7, 8]. Interestingly, Mor et al. have demonstrated that plant can serve as an expression host for wild-type BChE [7, 8] and its mutants [9, 14]. It has also been known that the plant-expressed BChE (pBChE) or a pBChE mutant has a relatively shorter half-life compared to the same protein expressed in CHO cells due to the lack of complete polysialylation during the post-translational modification (glycosylation) in plant. Nevertheless, co-expression of pBChE with additional genes required for *in planta* protein sialylation can produce the desired pBChE with the overall glycosylation profile resembling the plasma-derived orthologue in order to have a much longer biological half-life [19].

The above background shows that plant protein expression is a truly valuable source of protein production for human BChE and its mutants (including CocH3) for practical use. However, it is unknown whether our most recently designed catalytic antibody analog CocH3-Fc [4], an Fc-fusion protein, can also be expressed in a plant. We are particularly interested in the Fc-fusion protein, because Fc portion of the fusion protein lends itself to easier purification using conventional protein A chromatography, potentially reducing the number of processing steps in the manufacture of CocH3-Fc at large scale. We also wanted to know whether pCocH3-Fc expressed in a plant has a significantly longer biological half-life than the unfused protein pCocH3 expressed in the same plant. Here we report the establishment of a *Nicotiana benthamiana* plant expression system for the production of pCocH3-Fc. In comparison with the unfused pCocH3, pCocH3-Fc can be expressed more efficiently under the same experimental conditions. This is the first report of a heterologous expression of an Fc-fused cocaine hydrolase or BChE or BChE mutant in plants.

## Methods

### Constructions for plant expression

**Fig. 1:**
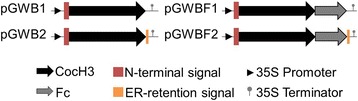
Diagram of constructs used in this study. CocH3: 1–529 amino acids of CocH3. Fc: Fc region of human IgG1. N-terminal ER-targeted signal: MDSKVTIICIRFLFWFLLLCMLIGKSHT. ER-retention signal: SEKDEL. Promoter: Cauliflower mosaic virus (CaMV) 35S promoter from pRTL2-GUS. Terminator: CaMV 35S terminator from pRTL2-GUS

Starting from the sequences of human BChE (accession number P06276 in the Swiss Protein Database), the CocH3 or CocH3-Fc cDNA was first subjected to codon optimization according to codon bias of *N. benthamiana*, and then cloned *via* Gateway (Invitrogen) cloning technology into the vector pSITE0A, a member of pSITE family *Agrobacterium* binary vectors developed by Dr. Goodin for the purpose of protein expression in plant [3]. As illustrated in Fig. 1, various constructions with or without a signal peptide, with or without fusion with Fc fragment were prepared *via* different combinations of corresponding primers: the coding sequences of amino acids 1–529 of the CocH3 were amplified with *pfu* DNA polymerase (Stratagene, La Jolla, CA) by using corresponding templates harboring point mutations A199S/F227A/S287G/A328W/Y332G on human BChE (stocked in this lab) with a forward primer 12-7_F_ENTR (for constructs without N-terminal signal peptide) or 12-7_F_sig_ENTR (for constructions with N-terminal signal peptide) and a reverse primer 12-7_R (for constructions without C-terminal signal peptide) or 12-7_R_sig (for constructions with C-terminal signal peptide). The Fc fragment was amplified by using a forward primer FC_F and a reverse primer FC_R_ENTR (for constructions without C-terminal signal peptide) or FC_R_sig _ENTR (for constructions with C-terminal signal peptide). All of the primers used are listed in Table 1. The CocH3-Fc fusion PCR was done based on 12 bp nucleotide sequences overlapping the 3’-end of CocH3 gene and 5’-end of Fc fragment, followed by a full-length PCR using a CocH3 forward primer and an Fc reverse primer. The promoter and terminator used are the cauliflower mosaic virus (CaMV) 35S promoter and the CaMV 35S terminator, respectively, from pRTL2-GUS [18].

**Table 1:** Primers used in this study

Primer	Sequence
12-7_F_ENTR	5′-CACCATGGAAGATGACATCATAATTGCAACA-3′
12-7_F_sig_ENTR	5′-CACCATGGATAGCAAAGTCACAATCATATG-3′
12-7_R	5′- ATAGAGCTCTTAGACTTTTGGAAAAAATGATGTCCAG-3′
12-7_R_sig	5′-ATTTCAGAGTTCATCCTTCTCAGAGAGACCCACACAACTTTCTTTCT-3′
FC_F	5′-TTCCAAAAGTCGTGGAGCCTAAGTCCTGCGACAA-3′
FC_R_ENTR	5′-ATTTTTACCCGGAGACAGGGAGAG-3′
FC_R_sig _ENTR	5′-ATTTCAGAGTTCATCCTTCTCAGATTTACCCGGAGACAGGGAGAG-3′

### Transient transfection of N. benthamiana with pCocH3 or pCocH3-Fc expression constructs

*N. benthamiana* wild-type plants used in this study were maintained by Dr. Michael Goodin’s lab in the College of Agriculture Greenhouse operations and facility at the University of Kentucky. The College of Agriculture Greenhouse operations and facility is administered by Facilities Management of the university. Young *N. benthamiana* plants with 4–5 true leaves were used for protein expression in this study. Expression plasmids (see Fig. 1) were transformed into *Agrobacterium tumefaciens* strain GV3850 following the standard freeze-thaw method [10]. After confirming the presence of plasmid by clone PCR, *A. tumefaciens* strains were cultured for overnight in 5 mL of LB media supplemented with spectinomycin (100 μg/mL), rifampicin (100 μg/mL), and streptomycin (20 μg/mL) on a shaker (200 rpm) at 28 °C, which by a ratio of 1:100 was transferred to fresh YEP medium (http://cshprotocols.cshlp.org/) supplemented with the same antibiotics. After OD_600_ reached ~0.7, *Agrobacterium* cells were collected by centrifugation at 4000 × *g* for 15 min at 4 °C, washed twice by using MES buffer (10 mM MgCl_2_, 10 mM MES, pH 5.6), and re-suspended in the MES buffer supplemented with 200 μM acetosyringone to OD_600_ = ~0.7. The cell suspension was kept at room temperature for 2–3 h, and then used to infiltrate plant leaves by vacuum infiltration. After keeping infiltrated plants in dark overnight, plants were cultured under light (14 h per day) at room temperature. Unless stated specifically otherwise, leaves were harvested at 3 days after infiltration, and immediately frozen in liquid nitrogen and kept at −80 °C for use in subsequent experiments.

### Protein extraction and purification

For small scale, leaves were grounded in liquid nitrogen to fine powder which was re-suspended in 100 mM phosphate buffer (PB) (pH 7.4) supplemented with protease inhibitor cocktail (ordered from Sigma) with a ratio of 3 mL buffer per gram leaves. For large scale, leaves were grounded by a blender (Oster) in the presence of cold PB buffer supplemented with the same protease inhibitor cocktail. For better plant cell lysis, the grounded leaf mixture was passed through a French Press (Thermo Scientific). After centrifuging at 23,000 × *g* for 15 min at 4 °C, the supernatant, after filtration through Miracloth (EMD Millipore), was obtained as the crude extract. The crude extract (containing pCocH3 or pCocH3-Fc) prepared from leaves was used for the initial enzyme activity assays (in triplicate).

Purification of the fusion protein pCocH3-Fc from the crude extract was conducted by using Protein A Fast Flow (GE Healthcare) affinity chromatography [24]. About 1 mL resin was first equilibrated with 20 CV (20 mL) of PB buffer, loaded with crude extract (~200 mL per 1 mL resin), and washed by 10–15 mL of PB buffer. Protein was eluted by 10 mL of 50 mM sodium citrate buffer (pH 4.5), and followed by another 10 mL of 50 mM sodium citrate buffer (pH 3.8). Eluted protein solutions were neutralized immediately with PB buffer and then concentrated by Amicon centrifuge tubes (Millipore).

Protein pCocH3 was not purified and CocH3 purified from CHO-expression system was used [24] for comparison in the kinetic analysis.

### Protein PEGylation

Protein conjugation with polyethylene glycol (PEG) polymer chains (PEGylation) was performed according to a manufacture-provided protocol (JenKem Technology) in order to extend the biological half-life of the protein. Briefly, the purified pCocH3-Fc protein (1 μg/μL) was quickly mixed with PEG 2000 at a ratio of 1:300 in 50 mM PB (pH 7.4), and incubated at 4 °C overnight. To remove the remaining PEG, protein preparations were filtered with Amicon Ultra-15 Centrifugal Filter Units (Millipore) with abundant amount of 100 mM PB, and finally concentrated to 500 μL.

### In vitro enzyme activity assay

To monitor the protein expression, the catalytic activity of the crude enzyme (pCocH3 or pCocH3-Fc) extract against cocaine was assayed rapidly by using [^3^H](−)-cocaine labeled on its benzene ring as described previously [17]. Reaction mixture (200 μL) containing 10 μL of enzyme solution, 50 μL of 400 nM cocaine containing 100 nCi [^3^H] -cocaine in 0.1 mM PB was incubated at 25 °C for 3 min, and stopped by adding 200 μL of 0.05 M HCl which neutralizes the labeled benzoic acid while ensuring a positive charge on the residual cocaine. [^3^H]-labeled benzoic acid was extracted by 1 mL of toluene and measured by scintillation counting. The radiometric data were analyzed as described previously [28].

The final kinetic characterization of pCocH3-Fc against cocaine was carried out on the purified pCocH3-Fc with our previously established protocol using the [^3^H](−)-cocaine [11, 24].

### Subjects for in vivo activity tests

Male CD1 mice (27–30 g) were obtained from Harlan (Indianapolis, IN) and housed in groups of 4–5 mice per cage. According to our previous experience, different strains of mice are expected to show very similar results for the effects of a given cocaine-metabolizing enzyme. CD1 mice generally have very visible tail veins, which makes the tail vein injection easier. For this reason, the same type of mice were used in our previous in vivo studies on the protein expressed in CHO cells [4]. All mice were allowed ad libitum access to food and water and were maintained on a 12-h light/dark cycle with lights on at 8:00 AM in a room kept at a temperature of 21–22 °C. Each mouse was used only once. Experiments were performed in the same colony room in accordance with the Guide for the Care and Use of Laboratory Animals as adopted and promulgated by the National Institutes of Health. The experimental protocol was pre-approved by the IACUC (Institutional Animal Care and Use Committee) at the University of Kentucky.

### Enzyme administration

Mouse was placed in a small restraint (TV-150 STD, Braintree Scientific, Inc., Braintree, MA) that left the tail exposed. The tail was wiped with an alcohol pad, then a 1-ml syringe with a 30-gauge needle (Becton, Dickinson and Company, Franklin Lakes, New Jersey) was used for infusion *via* a tail vein. The intravenous (IV) injection volume of the enzyme was controlled at 0.2 mL per 30 g of mouse body weight.

### Determination of the biological half-life in mice

Mice were injected IV (*via* the tail vein) with the enzyme (pCocH3 or pCocH3-Fc or its PEGylated form, denoted as PEG-pCocH3-Fc) in the dose of 0.075 mg/kg body weight. First, the saphenous vein was punctured with a needle. Approximately ~50 μL of blood sample was collected into a capillary tube at various time points after the enzyme injection. The blood samples were centrifuged at 5000 × *g* for 15 min. The isolated serum was tested for the enzyme activity as described above. The data for elimination of the enzyme from the circulation were fitted to a double-exponential equation as described previously [4].

### Protection experiment in mice

Mice were given 1 mg/kg pCocH3-Fc or saline intravenously (IV *via* tail vein), and then a lethal dose of cocaine (180 mg/kg) intraperitoneally (IP). Each mouse was monitored for its behavior and vitality for at least 1 h post injection. Mice injected with saline solution and cocaine were used as the negative control. Experiments were done in triplicate (*n* = 3 for each group).

## Results and discussion

### Expression of pCocH3-Fc

Expression constructs containing only a signal peptide to target expression through the secretory pathway (Fig. 1; pGWB1 and pGWBF1) did not result in any detectable protein expression following vacuum infiltration for up to 11 days. However, expression of constructs with ER-retention signal (Fig. 1; pGWB2 and pGWBF2) resulted in detectable enzyme activity 4 and 5 days post infiltration, suggesting that the protein expression was dependent upon the ER-retention signal.

**Fig. 2:**
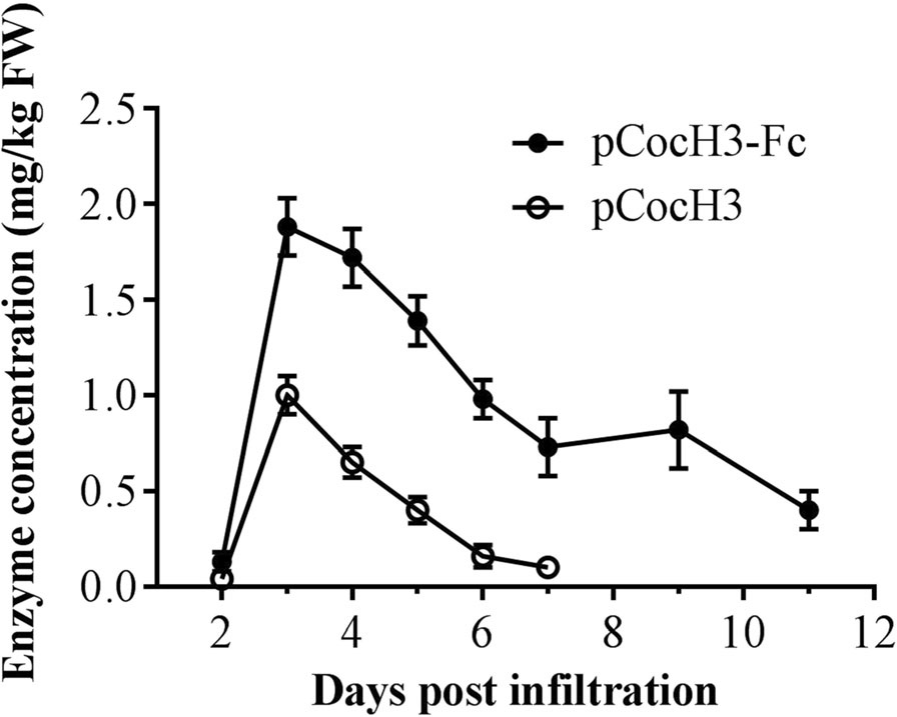
Expression profiles of pCocH3 and pCocH3-Fc. For each protein, three leaves per day were harvested from the same layer of three independent plants on the indicated time, and mixed together for enzyme extraction. The crude extracts prepared from leaves were used for the initial enzyme activity assays (in triplicate). The protein concentration was calculated based on the measured enzyme activity (V_max_) and the known catalytic parameter (*k*_cat_ = 5700 min^−1^)

Then, we checked the expression level in the plant leaves after the infiltration of agrobacteria by monitoring the cocaine-hydrolyzing activity of the crude extract. Both enzymes (pCocH3-Fc and pCocH3) were expressed rapidly in plant cells, and peaked on Day 3 after *Agrobacterium* infiltration; then, the cocaine-hydrolyzing activity in the plant leaves gradually went down. Depicted in Fig. 2 is a typical pattern of the enzyme expression in *N. benthamiana* leaves associated with fusion construct pCocH3-Fc. These results suggested that transient expression of the enzyme (pCocH3-Fc or pCocH3) in the plant was quite rapid and efficient, and that 3 days after bacteria infiltration should be the best time to harvest leaves. Our results are remarkably different from the earlier observation that the expression of human BChE or its mutant in *N. benthamiana* plant started at an extremely low level (<1 mg/kg) within the first eight (or more) days, but was able to continue and reached a peak after 14–17 days post *Agrobacterium* infiltration [14]. The remarkably different protein expression patterns are likely due to the difference between the vectors used. Specifically, the one used in the current study is a traditional non-replication vector which starts to diminish once it has entered plant leaves, whereas the one used in the earlier study [14] is a viral vector (ICON vector containing some viral functional components) able to replicate in plant cells. The viral vector is also able to communicate/travel between cells when a mobile protein is expressed.

It is notable that, under the same experimental conditions, the expression level of Fc-fused protein pCocH3-Fc was always higher than that of the corresponding protein pCocH3 by about two-fold. After the harvest time was determined to be 3 days, we further expressed pCocH3-Fc and pCocH3 in *N. benthamiana* leaves for multiple times. The replicate transient transfections yielded between 2 and 4 mg/kg FW for pCocH3-Fc with an average of ~3 mg/kg FW, while pCocH3 expression ranged between 1 and 2 mg/kg FW with an average of ~1.5 mg/kg FW. So, the Fc fusion approximately doubled the pCocH3 expression in *N. benthamiana* leaves under the same experimental conditions. For comparison, in our previous study [4] of the HSA-fused CocH1 (Albu-CocH1), the yield of the protein expression in the CHO cells after the transient transfection was between 1 and 2 mg per liter (L) of culture medium with an average of ~1.5 mg/L. So, the yield of the transient expression of pCocH3 in the plant is comparable to that of our previous transient expression of Albu-CocH1 in CHO cells. The average yield of the transient expression of pCocH3-Fc in *N. benthamiana* leaves is higher than that of the transient expression of Albu-CocH1 in CHO cells.

It should be pointed out that the average yield of ~3 mg/kg FW for pCocH3-Fc is still not high enough for large-scale production at low cost. It is desirable to have a yield of ~100 mg/kg FW (or more) feasible for large-scale production at low cost. So, it might be necessary to improve the yield by ~30-fold.

### In vitro activity of the purified enzyme

**Fig. 3:**
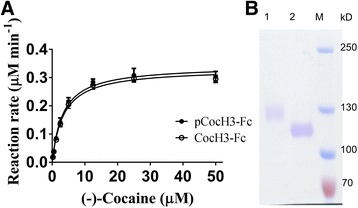
Kinetic and electrophoresis analyses of pCocH3-Fc and CHO cells-expressed CocH3-Fc. **a** In vitro kinetic profile of pCocH3-Fc and CocH3-Fc. The assays were carried out in triplicate. **b** SDS-PAGE in which lane M is associated with protein marker, lane 1 with CocH3-Fc expressed in CHO cells, and lane 2 with pCocH3-Fc

Fc fusion improved protein expression, but also facilitated protein purification. Very pure plant-expressed CocH3-Fc (pCocH3-Fc) protein (see Fig. 3) was obtained by using the convenient protein A affinity chromatography. However, purification of the unfused enzymes, such as wild-type pBChE, must undergo multiple steps of various chromatography [8, 14]. In this study, we purified Fc-fused CocH3, i.e. pCocH3-Fc, by using a one-step affinity chromatography, determined its steady-state kinetics against cocaine and compared it with CHO-expressed CocH3. According to the data depicted in Fig. 3a, we observed that *K*_M_ = 3.0 μM and *k*_cat_ = 5770 min^−1^ for pCocH3-Fc against cocaine; we did not note any significant change in the enzyme activity after the PEGylation (data not shown). In comparison, CHO cells-derived CocH3 showed a *K*_M_ of 3.1 μM and a *k*_cat_ of 5700 min^−1^, suggesting that pCocH3-Fc exhibited a similar catalytic activity against cocaine. These in vitro activity data are also similar to the earlier observation reported by Dr. Mor et al. [8] for the un-fused BChE mutant expressed in plant.

On the other hand, the SDS-PAGE data of the purified proteins also revealed that the pCocH3-Fc protein expressed in *N. benthamiana* leaves had a molecular weight significantly smaller than CocH3-Fc expressed in the CHO cells, as shown in Fig. 3b. The difference in molecular weight corresponds to the difference in glycosylation between the CHO and plant expression systems (no determination of glycosylation was made or reported in the paper). As well known, BChE from human plasma is a highly glycosylated protein, and each BChE molecule contains nine sugar chains which accounts for about 24 % of its total molecular weight [16]. The under-glycosylated BChE from an expression system other than mammalian cells was often a major hurdle for establishing a successful expression system of this enzyme, such as goat milk, insect cell and plant cell [1, 8, 12]. The under-glycosylation is expected to affect the pharmacokinetic profile of the protein, although it did not affect the in vitro enzyme activity.

### Biological half-life of pCocH3-Fc in mice

**Fig. 4:**
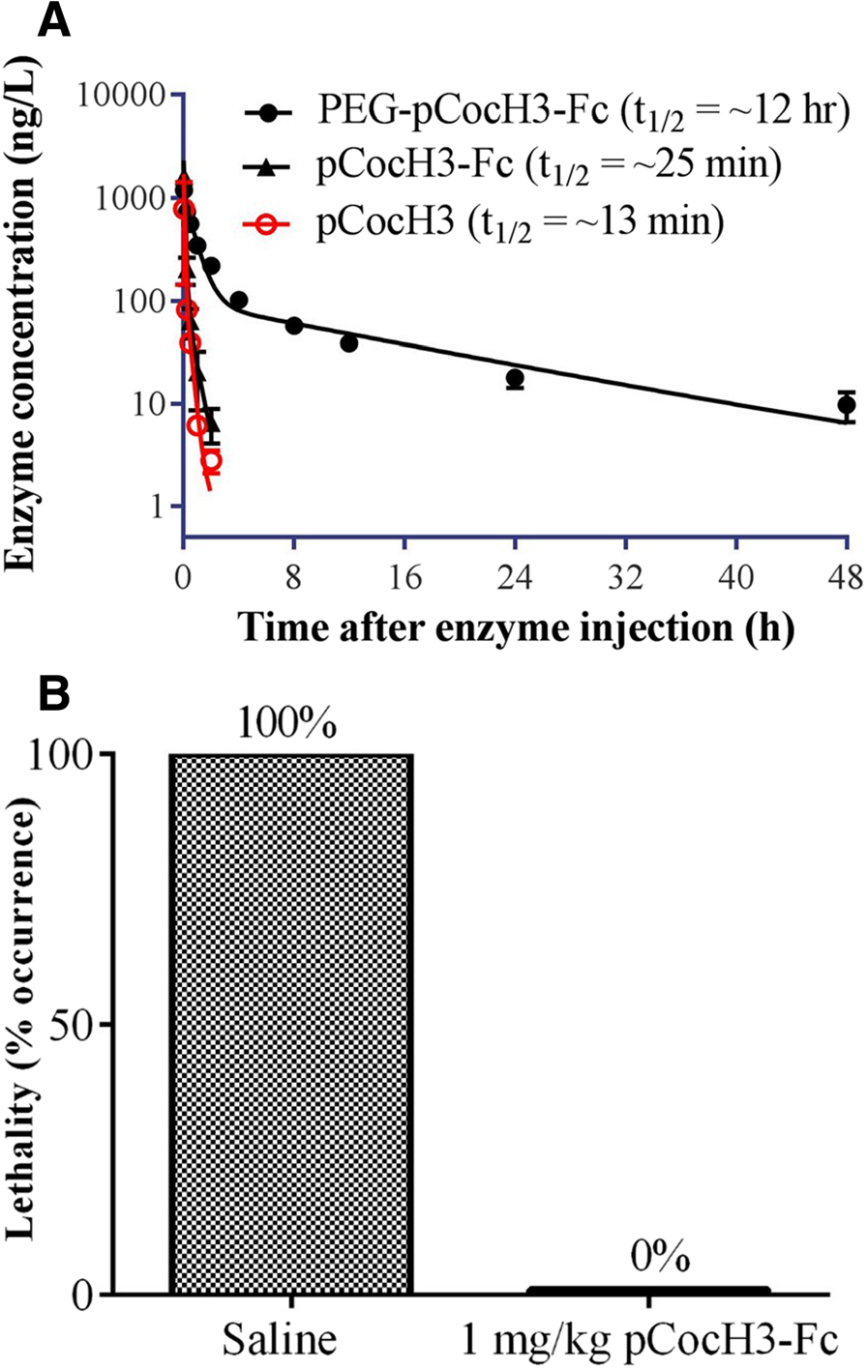
In vivo profiles of pCocH3-Fc. **a** Time-dependent concentrations of the active enzyme pCocH3-Fc or PEG-pCocH3-Fc (PEGylated pCocH3-Fc) in the plasma of mice after IV administration of the enzyme (0.075 mg/kg) determined in triplicate. **b** Lethality of 180 mg/kg cocaine (IP) with or without pretreatment of 1 mg/kg pCocH3-Fc. The lethality tests were performed in triplicate (*n* = 3 for each group). For the mice survived from the acute cocaine toxicity testing, the observation of animal behaviors was kept for at least 1 h to make sure that they continued to behave normally after the survival

The purified enzymes including pCocH3-Fc and the PEGylated pCocH3-Fc (denoted as PEG-pCocH3-Fc) were tested for their pharmacokinetic profiles in mice. Mice were administered IV with the purified enzyme (0.075 mg/kg). The blood was sampled at 2, 15, and 30 min, and 1, 2, 4, 8, 12, 24, and 48 h after the enzyme administration. Depicted in Fig. 4a are the time courses of the active enzyme (pCocH3-Fc or PEG-pCocH3-Fc or pCocH3) concentrations remained after the IV administration of the enzyme materials. The time-course of pCocH3 was determined by IV administration of the crude extract of pCocH3. For each enzyme sample, the enzyme concentrations ([E]) in the collected plasma samples were analyzed by detecting the enzyme activity (V_max_) of the plasma, resulting in [E] = V_max_ */k*_cat_. The same *k*_cat_ value (5700 min^−1^) was used for standardizing across samples.

The measured time-dependent concentrations of the active enzymes were fitted to a well-known double exponential equation (${\left[E\right]}_t=A{e}^{-{k}_1t}+B{e}^{-{k}_2t}$) which accounts for both the enzyme distribution process (the fast phase, associated with *k*_1_) and elimination process (the slow phase, associated with *k*_2_). The half-life associated with the enzyme elimination rate constant *k*_2_ is called the biological half-life *t*_1/2_ (the usually referred in vivo half-life). Thus, we obtained *t*_1/2_ = ~25 min for pCocH3-Fc and ~12 h for PEG-pCocH3-Fc, as depicted in Fig. 4. The biological half-life of pCocH3-Fc was only slightly longer than that (~13 min) of pCocH3 in mice.

### In vivo activity for cocaine detoxification

We further examined the potential therapeutic value of pCocH3-Fc by using an in vivo protection model [28] used previously for CocH3 expressed in mammalian cells. Specifically, we wanted to know whether pCocH3-Fc can effectively protect mice from the acute toxicity of a lethal dose of cocaine (180 mg/kg, LD_100_), because pretreatment with ~1 mg/kg CocH3 expressed in mammalian cells at 1 min before the cocaine injection fully protected the mice from such a lethal dose of cocaine [25, 28]. For the mice survived from the acute cocaine toxicity testing, the observation of animal behaviors was kept for at least 1 h to make sure that they continued to behave normally after the survival. As shown in Fig. 4b, for the negative control experiments (saline) without administration of the enzyme, IP injection of 180 mg/kg produced lethality in all mice tested. Pretreatment with 1 mg/kg pCocH3-Fc at 1 min before cocaine injection provided full protection in mice after receiving a lethal dose of cocaine (180 mg/kg, IP). So, the plant-expressed pCocH3-Fc was indeed active in vivo for cocaine detoxification.

## Conclusions

This is the first report on plant expression of the Fc fusion protein form of a cocaine hydrolase or BChE or BChE mutant, with the goal to know whether the promising CocH3-Fc can be expressed in a plant in any yield. The vector used in the present study might not be advanced enough, but it has been demonstrated that the active pCocH3-Fc enzyme can be expressed in plant *N. benthamiana* leaves with an average yield of ~3 mg/kg (compared to the average yield of ~1.5 mg/kg FW for the transient expression of pCocH3 in *N. benthamiana* leaves and the average yield of ~1.5 mg/L for the transient expression of Albu-CocH1 in CHO cells), and that the plant-expressed enzyme (pCocH3-Fc) was as active as the same enzyme (CocH3-Fc) expressed previously in CHO cells. In addition, the Fc fusion increased the yield of the enzyme expression in plant under the same experimental conditions, and greatly simplified purification procedure of the enzyme. But it might be necessary to further improve the yield by ~30-fold before the plant protein expression is feasible for large-scale production at low cost.

Further, it has been known that CocH3-Fc expressed in CHO cells has a considerably prolonged biological half-life than CocH3 expressed in CHO cells [4]. However, when the proteins were expressed in the plant, the biological half-life of pCocH3-Fc in mice was as short as that of pCocH3. Combination of the results obtained from the present study and the earlier studies showing a considerably improved biological half-life of the plant-expressed BChE [19] suggests that the *in planta* protein sialylation is essential for protein pBChE or pBChE mutant to have a long biological half-life, whether the protein is fused with Fc or not. In lack of the *in planta* protein sialylation in the present study, the Fc fusion did not prolong the biological half-life of the enzyme pCocH3 at all. Hence, further development for the Fc fusion protein production in plant should utilize a more advanced vector with additional genes required for the *in planta* protein sialylation [19]. It is expected that utilization of the more advanced vector would enable to more efficiently produce pCocH3-Fc with the overall glycosylation profile resembling the plasma-derived orthologue. The pCocH3-Fc protein with an improved glycosylation profile is expected to have a much longer biological half-life. Additional genes and proper promoters should also be included in the vector for a higher yield of the enzyme expression.

## Abbreviations

BChE: Butyrylcholinesterase
CHO: Chinese hamster ovary
CNS: Central nervous system
CocH: Cocaine hydrolase
CocH1: Cocaine hydrolase 1
CocH3: Cocaine hydrolase 3
CocH3-Fc: Cocaine hydrolase 3 fused with Fc region of human immunoglobulin G1
ER: Endoplasmic reticulum
Fc: fragment crystallizable (Fc) region of human immunoglobulin G1
HSA: Human serum albumin
IgG1: Immunoglobulin G1
pBChE: plant-expressed BChE protein
pCocH3: plant-expressed CocH3 protein
pCocH3-Fc: Plant-expressed CocH3-Fc protein
PEG-pCocH3-Fc: PEGylated pCocH3-Fc

## References

[1] Brazzolotto X, Wandhammer M, Ronco C, Trovaslet M, Jean L, Lockridge O, Renard PY, Nachon F (2012). Human butyrylcholinesterase produced in insect cells: huprine-based affinity purification and crystal structure. FEBS J.

[2] Brimijoin S, Gao Y, Anker JJ, Gliddon LA, LaFleur D, Shah R, Zhao Q, Singh M, Carroll ME (2008). A cocaine hydrolase engineered from human butyrylcholinesterase selectively blocks cocaine toxicity and reinstatement of drug seeking in rats. Neuropsychopharmacology.

[3] Chakrabarty R, Banerjee R, Chung SM, Farman M, Citovsky V, Hogenhout SA, Tzfira T, Goodin M (2007). PSITE vectors for stable integration or transient expression of autofluorescent protein fusions in plants: probing Nicotiana benthamiana-virus interactions. Mol Plant Microbe Interact.

[4] Chen X, Xue L, Hou S, Jin Z, Zhang T, Zheng F, Zhan C-G (2016). Long-acting cocaine hydrolase for addiction therapy. Proc Natl Acad Sci U S A.

[5] Cohen-Barak O, Wildeman J, van de Wetering J, Hettinga J, Schuilenga-Hut P, Gross A, Clark S, Bassan M, Gilgun-Sherki Y, Mendzelevski B, Spiegelstein O (2015). Safety, Pharmacokinetics, and Pharmacodynamics of TV-1380, a Novel Mutated Butyrylcholinesterase Treatment for Cocaine Addiction, After Single and Multiple Intramuscular Injections in Healthy Subjects. J Clin Pharmacol.

[6] Ersche KD, Jones PS, Williams GB, Turton AJ, Robbins TW, Bullmore ET (2012). Abnormal brain structure implicated in stimulant drug addiction. Science.

[7] Geyer BC, Kannan L, Cherni I, Woods RR, Soreq H, Mor TS (2010). Transgenic plants as a source for the bioscavenging enzyme human butyrylcholinesterase. Plant Biotechnol J.

[8] Geyer BC, Kannan L, Garnaud PE, Broomfield CA, Cadieux CL, Cherni I, Hodgins SM, Kasten SA, Kelley K, Kilbourne J, Oliver ZP, Otto TC, Puffenberger I, Reeves TE, Robbins N, Woods RR, Soreq H, Lenz DE, Cerasoli DM, Mor TS (2010). Plant-derived human butyrylcholinesterase, but not an organophosphorous-compound hydrolyzing variant thereof, protects rodents against nerve agents. Proc Natl Acad Sci U S A.

[9] Geyer BC, Woods RR, Mor TS (2008). Increased organophosphate scavenging in a butyrylcholinesterase mutant. Chem Biol Interact.

[10] Hofgen R, Willmitzer L (1988). Storage of competent cells for Agrobacterium transformation. Nucleic Acids Res.

[11] Hou S, Xue L, Yang W, Fang L, Zheng F, Zhan C-G (2013). Substrate selectivity of high-activity mutants of human butyrylcholinesterase. Org Biomol Chem.

[12] Huang YJ, Huang Y, Baldassarre H, Wang B, Lazaris A, Leduc M, Bilodeau AS, Bellemare A, Cote M, Herskovits P, Touati M, Turcotte C, Valeanu L, Lemee N, Wilgus H, Begin I, Bhatia B, Rao K, Neveu N, Brochu E, Pierson J, Hockley DK, Cerasoli DM, Lenz DE, Karatzas CN, Langermann S (2007). Recombinant human butyrylcholinesterase from milk of transgenic animals to protect against organophosphate poisoning. Proc Natl Acad Sci U S A.

[13] Landry DW, Zhao K, Yang GX, Glickman M, Georgiadis TM (1993). Antibody-catalyzed degradation of cocaine. Science.

[14] Larrimore KE, Barcus M, Kannan L, Gao Y, Zhan C-G, Brimijoin S, Mor T (2013). Plants as a source of butyrylcholinesterase variants designed for enhanced cocaine hydrolase activity. Chem Biol Interact.

[15] Milton AL, Everitt BJ (2012). Wiping drug memories. Science.

[16] Nicolet Y, Lockridge O, Masson P, Fontecilla-Camps JC, Nachon F (2003). Crystal structure of human butyrylcholinesterase and of its complexes with substrate and products. J Biol Chem.

[17] Pan Y, Gao D, Yang W, Cho H, Yang G, Tai H-H, Zhan C-G (2005). Computational redesign of human butyrylcholinesterase for anticocaine medication. Proc Natl Acad Sci U S A.

[18] Restrepo MA, Freed DD, Carrington JC (1990). Nuclear transport of plant potyviral proteins. Plant Cell.

[19] Schneider JD, Castilho A, Neumann L, Altmann F, Loos A, Kannan L, Mor TS, Steinkellner H (2014). Expression of human butyrylcholinesterase with an engineered glycosylation profile resembling the plasma-derived orthologue. Biotechnol J.

[20] Shram MJ, Cohen-Barak O, Chakraborty B, Bassan M, Schoedel KA, Hallak H, Eyal E, Weiss S, Gilgun Y, Sellers EM, Faulknor J, Spiegelstein O (2015). Assessment of pharmacokinetic and pharmacodynamic interactions between albumin-fused mutated butyrylcholinesterase and intravenously administered cocaine in recreational cocaine users. J Clin Psychopharmacol.

[21] Skolnick P, White D, Acri JB (2015). Editorial: emerging targets for stimulant use disorders: where to invest in an era of constrained resources?. CNS Neurol Disord Drug Targets.

[22] Sun H, Pang Y-P, Lockridge O, Brimijoin S (2002). Re-engineering butyrylcholinesterase as a cocaine hydrolase. Mol Pharmacol.

[23] Willyard C (2015). Quest for the quitting pill. Nature.

[24] Xue L, Hou S, Tong M, Fang L, Chen X, Jin Z, Tai H-H, Zheng F, Zhan C-G (2013). Preparation and in vivo characterization of a cocaine hydrolase engineered from human butyrylcholinesterase for metabolizing cocaine. Biochem J.

[25] Xue L, Ko M-C, Tong M, Yang W, Hou S, Fang L, Liu J, Zheng F, Woods JH, Tai H-H, Zhan C-G (2011). Design, preparation, and characterization of high-activity mutants of human butyrylcholinesterase specific for detoxification of cocaine. Mol Pharmacol.

[26] Yang W, Xue L, Fang L, Chen X, Zhan C-G (2010). Characterization of a high-activity mutant of human butyrylcholinesterase against (−)-cocaine. Chem Biol Interact.

[27] Zheng F, Xue L, Hou S, Liu J, Zhan M, Yang W, Zhan C-G (2014). A highly efficient cocaine-detoxifying enzyme obtained by computational design. Nat Commun.

[28] Zheng F, Yang WC, Ko MC, Liu JJ, Cho H, Gao DQ, Tong M, Tai HH, Woods JH, Zhan C-G (2008). Most efficient cocaine hydrolase designed by virtual screening of transition states. J Am Chem Soc.

[29] Zheng F, Zhan C-G (2012). Are pharmacokinetic approaches feasible for treatment of cocaine addiction and overdose?. Future Med Chem.

